# Evaluation of the Major Seed Storage Proteins, the Conglutins, Across Genetically Diverse Narrow-Leafed Lupin Varieties

**DOI:** 10.3389/fnut.2022.842168

**Published:** 2022-05-13

**Authors:** Arineh Tahmasian, Angéla Juhász, James A. Broadbent, Mitchell G. Nye-Wood, Thao T. Le, Michelle L. Colgrave

**Affiliations:** ^1^Australian Research Council Centre of Excellence for Innovations in Peptide and Protein Science, School of Science, Edith Cowan University, Joondalup, WA, Australia; ^2^Commonwealth Scientific and Industrial Research Organisation Agriculture and Food, St. Lucia, QLD, Australia; ^3^Department of Food Science and Microbiology, Auckland University of Technology, Auckland, New Zealand

**Keywords:** narrow-leafed lupin, *Lupinus angustifolius*, plant-based protein, conglutin, legume, proteomics, LC-MRM-MS

## Abstract

Lupin seeds have an excellent nutritional profile, including a high proportion of protein and dietary fiber. These qualities make lupin seeds an ideal candidate to help meet the growing global demand for complementary sources of protein. Of consequence to this application, there are nutritional and antinutritional properties assigned to the major lupin seed storage proteins—referred to as α-, β-, δ- and γ-conglutins The variation in the abundance of these protein families can impact the nutritional and bioactive properties of different lupin varieties. Hence, exploring the conglutin protein profiles across a diverse range of lupin varieties will yield knowledge that can facilitate the selection of superior genotypes for food applications or lupin crop improvement. To support this knowledge generation, discovery proteomics was applied for the identification of the 16 known conglutin subfamilies from 46 domestic and wild narrow-leafed lupin (NLL) genotypes. Consequently, the diversity of abundance of these proteins was evaluated using liquid chromatography–multiple reaction monitoring-mass spectrometry (LC–MRM-MS). This comparative study revealed a larger variability for the β- and δ-conglutin content across the lines under study. The absence/lower abundance of the β2- to β6-conglutin subfamilies in a subset of the domesticated cultivars led to substantially lower overall levels of the allergenic β-conglutin content in these NLLs, for which the elevation of the other conglutin families were observed. The diversity of the conglutin profiles revealed through this study—and the identification of potential hypoallergenic genotypes—will have great significance for lupin allergic consumers, food manufactures as well as grain breeders through the future development of lupin varieties with higher levels of desirable bioactive proteins and lower allergen content.

## Introduction

Interest in plant-based protein sources is on the rise (1) with growth driven by a rapidly increasing population, consumer demand and a groundswell for planetary health (2). The nutrient-dense grain legumes, also known as pulses, are one of the most promising plant-based complementary sources of protein, which could have a major contribution in enhancing global food security and environmental sustainability (3). Among the legumes, the lupin seeds stand out, owing to their favorable nutritional profiles associated to the remarkably high levels of quality protein and dietary fiber, as well as the nutraceutical benefits, including obesity-, type 2 diabetes-, and cardiovascular disease prevention (4).

Lupins belong to the diverse *Lupinus* genus of the Fabaceae family, which have been cultivated for thousands of years. Currently, narrow-leafed lupin (NLL, *Lupinus angustifolius)*, white lupin (*Lupinus albus*), pearl lupin (*Lupinus mutabilis*) and yellow lupin (*Lupinus luteus*) are the most cultivated species of lupin and are mainly grown as green manure in rotation with cereal crops or used as stockfeed. Despite their vast potential as a plant-based protein source, this ancient legume is under-utilized as a food ingredient; however, with the increasing demand in nutritious plant-based protein sources, the lupin market has the potential to expand beyond animal feed applications. NLL, also known as Australian sweet lupin, is one of the most recently domesticated crops (5). Genetic diversity studies targeting the NLL germplasm have resulted in the identification of a wide genetic and adaptive diversity, which can be accessed for improving lupin crops (6). In contrast, measurements of variation in the proteome of NLL germplasm have been hitherto underexplored.

The conglutin seed storage proteins are the most abundant protein class in NLL seeds, which not only serve as a nutrient reservoir for the germinating seed but also impact the nutritional quality of lupin seeds as a protein source for humans and livestock. These proteins have been classified into four major families: α-, β-, γ-, and δ- conglutins, which comprise three, seven, two, and four subfamilies, respectively (7). Distinct nutritional and nutraceutical properties have been attributed to each conglutin family; for example, the γ-conglutins are known to have blood glucose lowering effects (8), whilst β-conglutin proteins—which have demonstrated anti-inflammatory and antioxidant activities—are reported as the major proteins responsible for lupin allergy (9). Hence, variation in abundance of these proteins across different lupin varieties can impact their nutritional quality and bioactive effects. In fact, comparative evaluations have identified substantial variations in the functional properties (10), health benefits (11), allergenicity potential (12, 13) and digestibility (14) across different lupin cultivars. The relationships of the conglutin profiles with these traits are yet to be explored. The previous effort to study the NLL intraspecific proteome diversity have included a qualitative evaluation of the Australian domesticated cultivars using the polymorphism in the protein mass peak profiles (15), as well as the qualitative assessment of the four major conglutin families across a limited set of NLL cultivars (16). Still, there remains an opportunity to expand current knowledge regarding conglutin abundance in a wider variety of domesticated cultivars and diverse wild accessions to unravel the natural proteome diversity in lupin seeds and exploit this knowledge for crop improvement.

In this study, the diversity of the conglutin profiles across a panel of 46 genetically diverse NLL genotypes (including 16 domesticated and 30 wild accession) were assessed through a combination of discovery and targeted proteomics measurements. The detected conglutin peptides were used to evaluate the conservation of the conglutin protein sequences across the analyzed NLLs. The differentiation and quantitative study of the 16 known conglutin proteins across these genotypes were achieved through monitoring a set of marker peptides deemed representative of each conglutin subfamily. The knowledge gained from this work will facilitate progress in the identification of genotypes containing higher levels of desirable nutritional, and lower content of antinutritional, conglutins. The resulting varieties can be expected to produce seeds tailored for heath and food purposes or be incorporated in NLL breeding programs to further enhance the quality of the lupin grain as a food ingredient.

## Materials and Methods

### Plant Material

Overall, 46 NLL accessions were included in this study. This panel included: (a) 30 genetically diverse wild accessions, representing the natural geographic range of the species throughout the Mediterranean Basin (17, 18); and (b) 16 fully domesticated and semi-domesticated NLL accessions which comprise of 11 cultivars released throughout the four different phases of Australian lupin breeding program, two breeding lines (Australian and Belarusian) as well as three Polish cultivars. The seed material for these genotypes were obtained from the Australian Grains Genebank (Horsham, VIC, Australia), CSIRO Agriculture and Food (Floreat, WA, Australia) and Australian Grain Technologies (Northam, WA, Australia). The identifiers used for these accessions throughout the manuscript, as well as the information on their country of origin, domesticated/wild status and supplier are summarized in Supplementary Table 1. All the seed samples were inspected to exclude foreign contamination and ground into a fine powder using a mixer mill (model MM400 Retsch, Germany).

### Chemicals, Enzymes, and Solvents

All the reagents and chemicals used were of analytical grade. Tris-hydroxymethyl aminomethane hydrochloride (Tris-HCl), dithiothreitol (DTT), iodoacetamide (IAM), n-hexane, ammonium bicarbonate, acetonitrile (ACN), and dimethyl sulfoxide (DMSO) were from Sigma-Aldrich (Bayswater, VIC, Australia). Urea and formic acid were acquired from ChemSupply (Gillman, SA, Australia). Sequencing grade trypsin and protease inhibitor cocktail was purchased from Promega (Alexandria, NSW, Australia). BCA Protein Assay Kit Reducing Agent Compatible was obtained from Thermo Fisher Scientific (Scoresby, VIC, Australia). The HPLC-grade water (18 MΩ cm) was prepared using an Arium^®^ pro ultrapure water system (Sartorius, Gottingen, Germany).

### Sample Defatting

For lipid removal from the lupin samples, 200 μL of *n*-hexane solvent was added to micro-tubes containing ∼20 mg of lupin flour and vortexed until thoroughly combined. The samples were further mixed using a thermoshaker (model GRA13687717, Thermo Fisher Scientific, Scoresby, VIC, Australia) at 1,000 rpm for 20 min. This was followed by centrifugation (10 min) at 20,800 × g (with all steps repeated three times). Subsequently, the samples were air dried in a fume hood and stored at 3°C for protein extraction (which was conducted within 24 h).

### Protein Extraction and Protein Estimation

A recently optimized urea-based protocol was used for the efficient extraction of the proteins from lupin seeds (19). Briefly, 800 μL of urea buffer (8 M urea, 2% (w/v) DTT, 1% (v/v) protease inhibitor cocktail in 0.1 M Tris-HCl; pH 8.2) was added to 20 mg of defatted lupin varieties (n = 3). The mixture was vigorously vortexed and incubated in a thermoshaker (600 rpm) at RT for 45 min. The solutions were centrifuged for 15 min at 20,800 × g and aliquots of the supernatant were collected for subsequent analysis. Protein estimations were conducted using a BCA Protein Assay Kit (Pierce*™*, Thermo Fisher Scientific) according to the manufacturer’s protocol.

### Tryptic Digestion

The tryptic peptides were generated using the filter-aided sample preparation (FASP) procedure as described previously (20) with minor modifications. In brief, 25 μL of the protein extracts were transferred onto 10 kDa molecular weight cut-off (MWCO) filters (Millipore, Bayswater, VIC, Australia) and washed twice using 100 μL of urea buffer (8 M urea in 0.1 M Tris-HCl, pH 8.2) with centrifugation (20,800 × g, 10 min). After which 100 μL of 25 mM iodoacetamide (in 8 M urea in 1 M Tris-HCl) was added for cysteine alkylation and the samples incubated in the dark at RT for 20 min. The excess iodoacetamide was then removed through two consecutive washes (100 μL of urea buffer) and centrifugation (20,800 × g, 10 min) steps. This was followed by buffer exchange with two 100 μL volumes of 100 mM ammonium bicarbonate (pH = 8.4) and centrifugation (20,800 × g, 10 min). Subsequently, 125 μL of 0.02 μg/μL sequencing grade trypsin (in 50 mM ammonium bicarbonate) was added to each filter and the samples were incubated overnight at 37°C. The digested peptides were collected in fresh centrifuge tubes with centrifugation at 20,800 × g for 15 min. The filters were washed with 200 μL 0.1% formic acid and the combined filtrates were evaporated to dryness in a vacuum centrifuge.

### Global Proteome Measurement

The tryptic peptides were resuspended in 50 μL of 0.1% formic acid and the pooled samples of biological replicates were subjected to liquid chromatography-tandem mass spectrometry (LC–MS/MS), using an Ekspert nanoLC415 (Eksigent, Dublin, CA, United States) coupled to a TripleTOF 6600 MS (SCIEX, Redwood City, CA, United States) system. The detailed LC–MS acquisition method parameters were described precisely by Colgrave et al. (21). In summary, the peptide samples (6 μL) were desalted on a polar C18 ProteCol trap column (Trajan; 3 μm Particle Size x 300 Å Pore Size, 10 mm x 300 μm ID) with 0.1% formic acid for 5 min at a flow rate of 10 μL/min, then separated at flow rate of 5 μL/min on a ChromXP C18 (3 μm, 120 Å, 150 mm × 0.3 mm) column (30°C) by applying the following gradient: 5–45% B over period of 40 min, 45–90% B in 5 min, 5 min hold at 90% B, return to 5% B over 1 min and 11 min re-equilibration. Solvent A consisted of aqueous 5% DMSO and 0.1% formic acid, whilst solvent B consisted of aqueous 5% DMSO, 90% acetonitrile, and 0.1% formic acid.

The eluent from HPLC was introduced to the DuoSpray ion source of the mass spectrometer. The ion spray voltage floating was set to 5,500 V; the curtain gas to 30 psi; ion source gas 1 and 2–18 and 20 psi, respectively; and the heated interface was set at 100°C. The data was acquired in information dependent acquisition (IDA) mode, where the MS1 scan range was defined between 350 and 1,250 m/z using an accumulation time of 0.25 s. The 30 most intense precursors meeting the selection criteria of charge state between 2 and 5 and intensity greater than 150 were selected for further fragmentation (MS/MS). MS2 spectra were acquired over the mass range of 100–1,800 m/z with 0.05 s accumulation time. For optimum peptide fragmentation the manufacturer’s rolling collision energy (CE) and a collision energy spread (CES) of 5 V was applied. The dynamic exclusion was enabled to exclude precursor ions after two occurrences within a 15 s interval and a mass tolerance of 100 ppm (peaks within 4 Da of the precursor m/z were excluded).

### Lupin-Specific Database Creation

The *de novo* transcriptome assemblies of Tanjil, Unicrop and P27255 released by CSIRO (22), were retrieved from the Lupin Genome Portal.^1^ Open reading frames with a minimum of 150 nucleotide length cut-off value were predicted from the RNA-seq data. These sequences were translated and functionally annotated using Pfam domain analysis within the CLC Main Workbench v21.0.4 (23), which applies the hmmsearch algorithm (from the HMMER3 package version 3.1b1) for domain identification in proteins. These sequences were appended to the UniProt *Lupinus* protein sequence entries (32,833 sequences downloaded on 21/04/2020), CSIRO NLL protein database (see text footnote 1), Biognosys iRT pseudo-protein sequence as well as the common repository of adventitious proteins (cRAP). Subsequently, the sequences with 100% identity were excluded from the final database (63,046 sequences).

### Prediction of Potential Conglutin Sequences, Phylogenetic Analysis, and Epitope Mapping

To identify the potential conglutin isoforms within the database, the 16 reported NLL conglutin sequences (referred to as reference conglutins throughout the manuscript) were searched against the lupin database, using the BLASTp algorithm in CLC Main Workbench v21.0.4. For discrimination of the conglutin sub-types the sequences with minimum 75% sequence identity with reference conglutins were subjected to homology searching (BLASTp) in NCBI, the resulted conglutin hits were additionally confirmed by the evaluation of the distinctive structural features such as the conserved Pfam domains and cysteine patterns. The known and predicted conglutin sequences were aligned using the Clustal W algorithm (24) and the corresponding phylogenetic tree for each conglutin family was constructed within the CLC Main Workbench v21.0.4. For identification of the potential allergenic regions in conglutin sequences, the list of the known epitopes was retrieved from the Immune Epitope Database and Analysis Resource website^2^ and used as an input for a motif search (100% sequence identity) within the CLC Main Workbench v21.0.4.

### Protein Identification

ProteinPilot 5.0.3 software (SCIEX) with the Paragon and ProGroup algorithms was used to search the raw MS (IDA) data against the *in silico* tryptic digests of the custom-built lupin database (25). The search effort was set as “Thorough ID” with iodoacetamide as cysteine alkylation agent and trypsin as digestion enzyme; TripleTOF 6600 was selected for the instrument type and biological modifications were enabled for ID focus. The SCIEX FDR analysis tool was used for estimation of false discovery rate (FDR) (26). A database search combining all IDA files was conducted to attain a complete list of proteins identified from all the lupin varieties; the reported identification yields represent data at a 1% global FDR cut-off. The protein identifications were robustly aligned across the group files using the SCIEX Protein Alignment Template v3.002p.

### Conglutin-Derived Peptide Mapping

A custom R script was used to filter the conglutin-derived tryptic peptides meeting a 1% FDR threshold with no unusual modifications (allowing carbamidomethyl Cys, oxidation of Met, and pyroglutamination of N-terminal Gln) from the FDR reports. These peptides were then aligned across the NLL samples by means of an in-house Python script and classified into six groups based on their identification frequency, as follow: identified across (a) 1–5; (b) 6–15; (c) 16–25; (d) 26–35; (e) 35–40 and (f) 41–46 NLL accessions. Using the motif search tool in the CLC Main Workbench 21.0.4 software, the peptide sequences were mapped to the reference conglutin protein sequences using 100% sequence identity, color-coded based on the groups above and evaluated for defining the conserved/variable regions of these proteins among the study genotypes.

### Targeted Assay Development and Peptide Specificity Analysis

The conglutin proteins identified at 1% FDR from the combined database searching were imported into Skyline software (v19.1.0.193) and subjected to *in silico* digestion. The ProteinPilot group files from the discovery proteomics data collected from lupin samples previously in our laboratory (19) and the combined search from the present study were used to build a BiblioSpec library containing peptides with a confidence value of > 0.95 (1% FDR). The library matched tryptic peptides (allowing one missed cleavage) between 8 to 30 amino acids in length with no variable modifications retained for the preliminary analyses (278 peptides). The spectral library was employed to determine the optimal multiple reaction monitoring (MRM) transitions for each peptide (six transition per peptide) and this unscheduled method was used to acquire data using a pool of all replicates. The results from these analyses were used to schedule retention times and refine the peptide and MRM transition list (minimum three transitions per peptide). The final targeted method included 760 transitions and 250 peptides (including modified forms), representing 21 proteins (Supplementary Data Sheet 2).

For discerning the unique peptides for each conglutin protein, the specificity of the MRM peptides was investigated (on 2021/06/02) using the peptide match tool within the Protein Information Resource website, where the Tanjil database (accessed directly from UniProt) was set as the background proteome (27).

### Liquid Chromatography-Multiple Reaction Monitoring-Mass Spectrometry

The experimental samples were divided into two batches. To allow the post-acquisition removal of the systematic non-biological variance between the batches, the injection order of the samples was randomized. A QC sample composed of 1 μg/μL BSA tryptic digestion spiked with iRT peptides (1:10) was analyzed prior to the batch and periodically throughout the batch and was used to monitor the instrument performance and the batch effect.

The chromatographic separation of the tryptic peptides (4 μL) was achieved on an Exion AD UHPLC system (SCIEX), and the quantitative acquisition was performed with a 6500 + QTRAP mass spectrometer (SCIEX). The MRM data was acquired in positive ion mode with the ion source temperature and the ion spray voltage—floating set at 500°C and 5,500 V, respectively. The MRM transitions were scheduled to be monitored within 60 s of their expected retention time (± 30 s) and the cycle time was set at 0.6 s. Skyline software (v19.1.0.193) was utilized for peak integration (28), wherein all the MRM peaks were inspected manually to ensure correct peak detection, signal-to-noise (S/N) > 5, and accurate integration.

### Peptide Data Processing and Statistical Analysis

The peptide abundance data was exported from Skyline and the batch effect was corrected by using the “Remove batch effect” function within the *Limma* R package using the batch information and the sample injection order as the covariate (29). The technical variation across the triplicates of each genotype was assessed by examining the coefficient of variation (CV) for each peptide. In total, 82 MRM peptides specific to the conglutin subfamilies (which exhibited the highest signal intensity and the lowest technical variability) were selected for the evaluation of the conglutin protein profiles across the NLL genotypes.

The heatmap of the quantitative peptide data (log10 transformed) was generated in the Morpheus analysis software^3^ (Broad Institute, Cambridge MA, United States) where the one minus Pearson correlation metric was selected for conducting the unsupervised hierarchical cluster analysis (HCA). The multivariate relationships of the observations were assessed through principal component analysis (PCA) of the peptide level data (log10 transformed) within the SIMCA software (Sartorius Stedim Biotech, v15.0). For multiple comparisons of the experimental groups one-way ANOVA followed by Dunnett’s test was applied in GraphPad Prism v8 and the graphs comparing the conglutin profiles were generated using a custom R script.

## Results and Discussion

Lupin conglutins are the major seed storage proteins which are known to have many health-promoting attributes, for example blood glucose lowering (8) and enhanced satiety (30) effects. In spite of these attributes conglutin proteins have also shown some undesirable allergenic properties. In the present study, the qualitative and quantitative diversity of the conglutin proteins were evaluated across a wide range of domesticated and wild NLL accessions from 14 countries (Algeria, Australia, Belarus, Cyprus, France, Greece, Israel, Italy, Morocco, Poland, Portugal, Spain, Syria, and Turkey) using LC–MRM-MS. Screening the available *Lupinus angustifolius* germplasm will provide insight into the intraspecific proteome diversity and lead to identification of varieties that contain higher levels of preferential and lower amount of detrimental conglutins. The outcome of which can benefit lupin breeding programs in the development of varieties with an improved protein complement.

### Discovery of Potential Conglutin Sequences Within the Database

**FIGURE 1:**
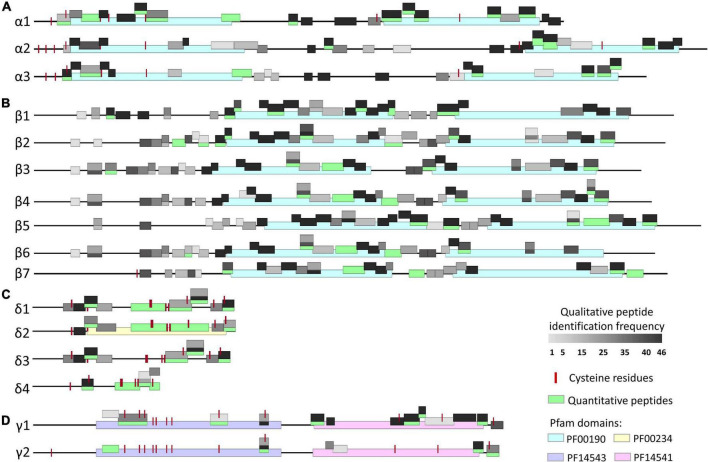
Schematic representation of the main structural findings and conserved regions in **(A)** α-, **(B)** β-, **(C)** δ-, and **(D)** γ-conglutin sequences, as well as the marker peptides used in LC-MRM-MS measurement of these proteins. The Pfam domains are highlighted in blue (Cupin_1), yellow (Tryp_alpha_amyl), purple (TAXi_N), and pink (TAXi_C). The red lines show the position of cysteine residues, and the green blocks are representative of MRM marker peptides (quantitative peptides). The conglutin peptides identified by IDA (qualitative peptides) are depicted by gray blocks, wherein the darker color indicates sequence regions commonly identified across the analyzed lines (white blocks, 1–5 cultivars; black blocks, 40–46 cultivars).

The lupin database used in this study was constructed using the proteomic, transcriptomic, and genomic resources available for different lupin varieties; consequently, it may include many inter-cultivar conglutin protein variants. Using bioinformatic techniques all the putative conglutin sequences present in the database were determined. To this end, the sequences with > 75% sequence identity with the 16 known NLL conglutin proteins were defined, and this repertoire was further constricted by evaluating the presence of discriminative structural features (such as conserved Pfam domains and cysteine patterns) in these sequences. This resulted in the identification of an additional 33 potential conglutin isoforms within the database, belonging to the four major α-, β-, δ-, and γ-conglutin families. The α-conglutins were recognizable by the presence of the conserved patterns of CX_n_C and two cupin_1 (PF00190) domains (between 36–201 and 405–552 residues) in their sequences (Figure 1A). The structural characteristics considered for distinguishment of the β-conglutins included the presence of two cupin_1 (PF00190) domains (between 180–330 and 390–551 residues) and lack of cysteine residues (Figure 1B), which were found with a specific pattern in other members of vicilin-like proteins (31). Moreover, two semi-conserved motifs (HYX_n_R and QQDEQEX_n_YX_n_LS) were noted in the globular domain of these proteins. The δ-conglutin proteins were distinct due to the existence of the conserved patterns of CX_n_CX_n_CCX_n_CXCX_n_CX_n_CX_n_C which is generally a characteristic of the prolamin superfamily proteins (32). Furthermore, a tryp_alpha_amyl domain (PF00234) was found in the δ2 protein structure (Figure 1C). Finally, the xylanase inhibitor N- and C-terminal domains (TAXi-N; PF14543 and TAXi-C; PF14541) between 62–237 and 269–430 residues were the main structural characteristic of the γ-conglutins where the cystine rich N-terminal region included conserved CX_n_C repeated patterns (Figure 1D).

### Protein and Peptide Identification

The IDA data for each NLL variety was searched against the lupin specific database. This resulted in variable protein and peptide identification yields at 1% FDR (between 1,418–1,994 proteins and 6,508–10,113 peptides) from these lines, the highest and the lowest protein identification yields were observed for D1 and W28 accessions, respectively (Supplementary Table 2). In addition to the individual searches a combined database search was conducted on the discovery proteomics datasets encompassing all the 46 NLLs, which led to the identification of 3,534 proteins and 20,371 peptides (1% FDR). The comparative qualitative analysis of the detected proteins across the analyzed lines, indicated that ∼14% of the identified proteome was commonly detectable across all the 46 NLL genotypes, whilst ∼5% the identifications were unique to an individual NLL variety. The substantially higher (>43%) identification yield achieved through the combined search compared to the individual searches, as well as the low proportion of the commonly identified proteome affirms a high level of proteome diversity among the analyzed lines. The evaluation of the aligned discovery metrics for the representation of the predicted conglutin proteins revealed identification evidence for the characterization of four α-, twelve β-, three δ-, and two γ-conglutin proteins based on unique peptide evidence. There are three α-conglutins known from NLL. The extra α-conglutin identification refers to a transcriptome translated sequence from *L. angustifolius* cultivar Unicrop which shares high sequence identity (96.1%) with α2-conglutin reference sequence (UniProt ID: F5B8V7). Based on the phylogenetic relationships of these proteins (Supplementary Figure 1A), this is likely to be a cultivar specific variant of the α2 isoform. Whereas the additional four β-conglutin hits (present in the UniProt NLL proteome database) exhibited a separate clustering from the known β-conglutins in the phylogenetic tree (Supplementary Figure 1B). However, the presence of other conglutin subfamilies in NLL has been suggested to be unlikely based on the in-depth cv Tanjil transcriptomic and draft genome sequence analyses (7). Furthermore, the identification of the δ1-conglutin protein was unachievable, which can be explained by the high sequence identity (> 98%) of this protein with the δ3 isoform. The 21 conglutin sequences identified based on unique peptide evidence were selected for targeted quantitative method development.

### Evaluation of Conglutin Protein Sequence Conservation Across the Analyzed Narrow-Leafed Lupin Lines

The alignment of the detected conglutin peptides across the samples was used to investigate the conglutin protein sequence conservation between the 46 NLL genotypes under study. In total, 244 conglutin peptides were detected within the samples of which 86 peptides (∼35%) were commonly identified across 41–46 NLL varieties, whilst 42 (∼17%) peptides were only detected in 1 to 5 NLL lines. The highest number of conglutin peptides (166) were identified from the Syrian W10 accession, followed by the Italian W1 (163 conglutin peptides) accession and the domesticated Australian D7 and D13 (162 conglutin peptides) cultivars. All conglutin peptides detected through the discovery proteomics (fully tryptic and containing no unusual modifications) and the MRM quantitative peptides were mapped onto the 16 reference conglutin sequences (Figures 1A–D). The highly conserved α- and β-conglutin peptides (identified in more than 40 lines) were mainly found on the two β-barrel structures of these sequences, which correspond to the cupin_1 domain regions in these proteins (Figures 1A,B). Notably, for β-conglutins, the least conserved peptides (present in less than 5 accessions) were mostly mapped on the first 200 N-terminal amnio acid residues of these sequences. This region—consisting of 8–10 α-helices—is known as the mobile N-terminal arm, which has been also shown to embrace the largest structural variability of the seven NLL β-conglutins (33). It has been suggested that the putative functional differences of the β-conglutin isoforms can be related to these structural differences (9). In the γ1-conglutin the peptide sequences that were identifiable across more than 40 lines were found within the 269–430 residues that correlates with the TAXi-C domain position within this protein (Figure 1D). Few peptides with high conservations were also identifiable within the δ2 to δ4 and γ2 sequences (Figures 1C,D).

### Selection of Marker Peptides for Each Conglutin Subfamilies

The conglutin-derived peptides identified by the combined database searching were selected for MRM-MS relative quantitation of these proteins. The peptides for each conglutin subfamily were selected using the following criteria: (1) specificity of the peptide sequence to the target protein (evaluated against Tanjil reference proteome); (2) fully tryptic; (3) identified with ≥ 95% confidence; (4) restricted modifications to oxidation (M), carbamidomethyl (C), and pyro-Glu (N-terminal Gln); and (5) a signal to noise ratio (S/N) > 5. For the accurate quantitation of the target proteins, the peptide list was further filtered to retain maker peptides with the highest peak signal intensity and the lowest technical variability for each conglutin protein; a total of 22, 37, 13, and 10 marker peptides were selected for evaluation of the α-, β-, δ-, and γ-conglutin proteins across 46 NLL genotypes. On the occasions where the measurement of an individual protein was unachievable (because of high sequence similarity and a lack of unique peptides), the quantified relative abundance of the peptides was allocated to a protein group. The list of marker peptides selected for the conglutin protein subclasses, their corresponding technical variance and the average peak areas are represented in Supplementary Table 3. Typically, technical variation related to sample preparation and analysis of < 13% was observed for the marker peptides (with the exclusion of five peptides).

### Multivariate Analysis of the Relative Abundance Data

**FIGURE 2:**
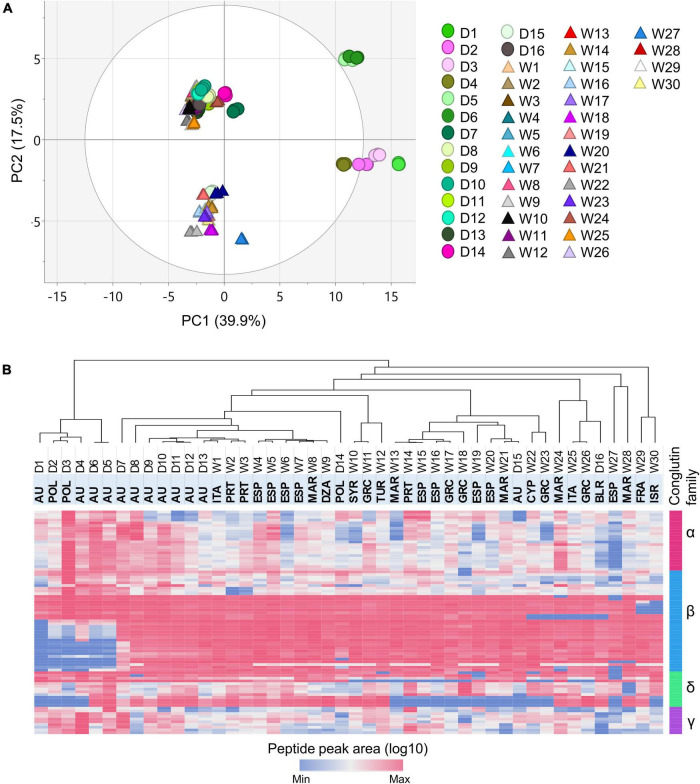
Multivariate analysis of the peptide level abundance data. **(A)** The PCA scores plot, PC1 vs. PC2, showing the separation of 46 NLL genotypes based on the conglutin-derived peptide data (log10). Each color represents an NLL genotype where the wild (triangles) and domesticated (circles) varieties are indicated. **(B)** The heatmap displaying the monitored marker peptide peak areas (log10) across the analyzed lines. The dendrogram from the unsupervised HCA illustrating the similarity of the NLL varieties in terms of conglutin abundance. Each column corresponds to a genotype and every row represents a peptide. The highlighted row at the top illustrates the counties of origin for the lines under study. The country names are abbreviated as follows: DZA (Algeria), AU (Australia), BLR (Belarus), CYP (Cyprus), FRA (France), GRC (Greece), ISR (Israel), ITA (Italy), MAR (Morocco), POL (Poland), PRT (Portugal), ESP (Spain), Syria (SYR), and TUR (Turkey).

Unsupervised principal component analysis (PCA) was applied to the peptide level abundance data (log10 scaled), to probe the relationships of the analyzed NLLs in terms of the conglutin protein contents. The PCA score plot revealed vivid differences in the conglutin profiles of the 46 lines (Figure 2A). The first and second principal components (PC1 and PC2, respectively) together explained ∼57.4% of the variability of the data, where PC1 (39.9%) effectively summarized the variance primarily related to the D1–D6 cultivars, and PC2 (17.5%) represented the differences mainly driven from W13-W17, W19-W23, W28, W31, and D15 accessions.

Evaluation of the loading vectors determined that the peptides assigned to the β 2-, β 3-, and β4,6- subfamilies with negative correlation in PC1, together with the δ2 and δ4 peptides—which correlated positively in PC2—led the separation across the accessions. Although distinct differences were observed across the domesticated cultivars based on their conglutin contents, no clear grouping was revealed based on the European or Australian origin of these NLLs. Previous genetic diversity studies provided concordant evidence regarding the close relationships of the Australian and European domesticated populations, which suggests shared ancestry or exchange of breeding materials (34).

The heatmap of the log10 transformed quantitative peptide data and the corresponding unsupervised hierarchical cluster analysis (HCA) revealed that the NLL lines fall into two major clusters based on the distribution of the conglutin peptides (Figure 2B). Therein, the stratification of the six domesticated lines (D1–D6) from the remaining accessions was clearly replicated. The Australian and Polish cultivars in this group demonstrated distinct conglutin protein profiles compared to the other 40 NLL accessions, the pedigree information available for the Australian NLL cultivars in this cluster (35) suggests a distant relationship among the D1, D4, D5, and D6 cultivars (relatively closer for D1 and D4). This indicates that the pedigree records solely are unable to explain the distinctiveness of the conglutin profiles of this cluster.

Within the second major cluster the Australian D7–D12 as well as the Polish D14 cultivated varieties, exhibited close grouping with a set of wild accessions mainly from the Iberian Peninsula (Spain and Portugal) and of North African (Morocco and Algeria) origins. The similarity of the conglutin profiles of these accessions can be ascribed to the western Mediterranean origin of the cultivated NLL germplasm (34). Contrarily, the more recent Australian D15 cultivar, which includes new wild ecotypes in its pedigree (36), appeared in a separate sub-group clustered with wild NLLs from Southern Europe (Greece and Cyprus), Iberian Peninsula (Portugal, Spain) and North Africa (Morocco). Moreover, within this major cluster the Belarussian D16 breeding line displayed a separate clustering from the other domesticated cultivars, the previous comparative diversity study of the available NLL varieties and breeding lines identified the Belarusian domesticated accessions genetically discrete, this was concluded to be a consequence of the complex wild crosses used in the Belarusian breeding program (34). Lastly, among the wild accessions under study, the W27–W30 accessions displayed the most distinct conglutin profiles.

### Comparative Quantitative Evaluation of the Conglutin Families Across the Analyzed Narrow-Leafed Lupin Accessions

**FIGURE 3:**
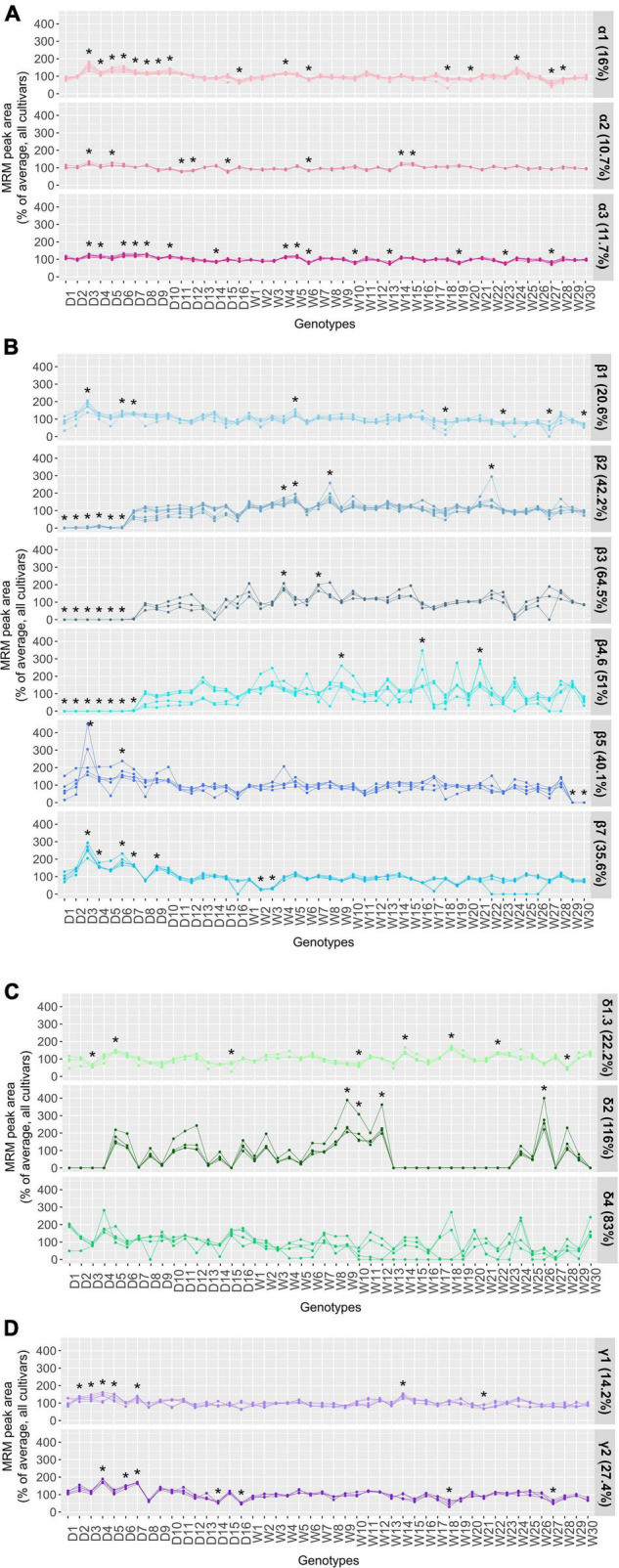
Relative quantitation of the marker peptides monitored for **(A)** α-, **(B)** β-, **(C)** δ-, and **(D)** γ-conglutin families. The line graphs in each panel compare the relative abundance of the peptides measured for each conglutin subfamily. For ease of comparison the MRM peak area of each peptide was converted to percentage relative to the average peak area for all accessions. One-way ANOVA with Dunnett test was conducted for the multiple comparisons of the experimental groups and determining the significant differences (*p < 0.05).

Distinctive biological functions and nutritional properties have been ascribed to the four major conglutin families. The comparative quantitative studies of these proteins across the NLL resources available, in addition to its significance for crop improvement, can also provide insight into the differing nutritional and health beneficial properties of the varieties available. Herein, the diversity of the major seed storage proteins across 46 NLLs was evaluated through comparing the abundances of the specific peptides monitored for each conglutin subfamily. To facilitate the comparison of different peptide abundance the raw peak peptide areas are converted into a percentage relative to the average peak area for all 46 NLL accessions and presented in a series of line graphs (Figures 3A–D). The overall levels of conglutin families were assessed using the summed peak area of the peptides belonging to each family, which was also normalized to the average percentage of total α-, β-, δ- and γ-conglutin contents across the analyzed lines (Supplementary Figures 3A–D). These analyses showed major differences in the abundance of the conglutin seed storage proteins. Results and discussion for each family follow below.

#### Alpha-Conglutin Protein Family

The α-conglutin family, also known as legumin-like or 11S globulin family, is the second most abundant protein class in lupin seeds and comprises three unique protein subfamilies, among which the α1 protein is more divergent in amino acid sequence (< 40% sequence identity) in comparison to the α2 and α3 proteins, which are closely related (∼70% identity). These proteins also have high homology with the α-conglutin sequences from distant lupin species and 11S globulins from other legumes (37). In fact, it has been highlighted that the α-conglutins are potentially associated with the immunologic cross-reactivity phenomenon with other legumes, particularly with the Ara h 3 peanut and Gly m 6 soybean allergens (38). The *in silico* investigation of the presence of known linear epitopes in the α-conglutin proteins identified Gly m 6 related epitopes, which were more frequently present in α1-conglutin (Supplementary Figure 2A). These epitopes were mainly found in the acidic subunit of these proteins which is a known immunoreactive fraction from lupin (13). The discrimination and the quantitation of the α 1-, α 2-, and α3-conglutins were achieved through monitoring the relative abundance of 11, 4, and 7 specific peptides, respectively. The α-subfamily specific peptides demonstrated similar patterns with small variations ranging from 11 to 16% across the NLLs examined (Figure 3A). The consistency of the α-conglutin protein levels across different NLL modern cultivars have been demonstrated previously (16); however, distinct α-conglutin RNA expression patterns were found between different lupin species (7). The Polish domesticated D3 cultivar with relatively high total protein content (22.8% above average; Supplementary Table 2) exhibited the highest α-conglutin levels and the Spanish W27 wild accession with comparatively lower total protein estimated value (22.4% lower than average; Supplementary Table 2) was found to have the lowest overall α-conglutin content (Supplementary Figure 3A).

#### Beta-Conglutin Protein Family

The vicilin-like β-conglutins, which belong to 7S globulin family, are the most abundant proteins in NLL seeds. These proteins are involved in multifunctional roles including seedling growth, plant development and antifungal defense response. They also display nutraceutical characteristics such as anti-inflammatory, hypoglycemic and antioxidant effects (9); however, anti-nutritional factors are also attributed to these proteins, which are characterized as the major allergens from lupin (in NLL named as Lup an 1; WHO/IUIS). Besides being involved in the primary lupin allergy the β-conglutins are also reported to be the target of immune system cross-reactivity from other legume allergens mainly peanut Ara h 1 and soybean Gly m 5 (38). The epitope mapping analysis revealed the presence of two soybean Gly m 5 epitopes, which were conserved across all the proteins of this family (Supplementary Figure 2B). These highly conserved sequence regions may have an involvement in the cross-reactivities observed for these legumes.

Seven protein subfamilies (β1 to β7) with relatively high amino acid sequence similarities (>75.6%) are included in the β-conglutin family, the quantitative evaluation of which was achieved through monitoring 3 to 8 quantitative peptides, across the 46 NLL accessions (Supplementary Table 3). Among the β-conglutin subfamilies the β4- and β6-conglutin proteins have the highest degree of sequence identity (97.8%). Specific peptides from β4 (GLTFPGSTEDVER and QLDTEVK) and β6 (QSAYER) proteins were identified, their relative abundance displayed similar patterns to the shared peptides (FGNFYEITPNR and ILLGNEDEQEDDEQR) from these two proteins (β4,6 group) across the 46 NLLs under study. This is suggestive of a common regulatory mechanism to produce these two highly similar proteins. A similar observation was also noted for the other β-conglutin subclasses; for instance, the proteins in the β2 group (including UniProt IDs: F5B8W0, A0A1J7FNI8, A0A1J7G5F6, A0A1J7GN79) have been quantitatively assessed using eight peptides, four of which are unique to F5B8W0 and the others are shared among these proteins; however, the trend of all peptides correlated across the analyzed NLL varieties. Altogether, larger biological differences (between 20.6 and 64.5%) were observed for the relative abundance of the β-conglutin protein subfamilies (Figure 3B); the variability was particularly notable for β2 to β7 conglutin subfamilies.

The distinct interspecific β-conglutin RNA expression patterns (7) and the presence of these proteins at different levels across a number of Australian domesticated cultivars (16) have been demonstrated previously. Herein, across the lower β-conglutin containing D1–D6 cultivars, the peptides monitored for β2, β3 and β4,6 protein subfamilies were either absent or present at significantly lower levels (∼99%). Similar β-conglutin peptide profiles were noted for D7 cultivar, apart from the higher abundant β2 peptides measured from this cultivar. In parallel, a comparative proteomics study of white lupin varieties also demonstrated the disappearance of a set of β-conglutins in the domesticated cultivar analyzed (39). This signifies the breeding strategies used during the domestication of these lines have led to development of lupin varieties with reduced β-conglutin content, which can potentially serve as hypoallergenic candidates for commercial cultivation or be exploited in breeding strategies to develop improved hypoallergenic lupin varieties. However, clinical trials will be required to determine if this degree of alteration in the β-conglutin profiles can prevent the adverse allergic reactions caused by lupin and/or influence the allergy-eliciting required dose.

Interestingly, among the wild accessions, the W27 with relatively low total protein estimate (∼23% lower than average; Supplementary Table 2) and smaller seed size, displayed a substantially reduced β-conglutin content, wherein all the β-conglutin subfamilies were present but downregulated. The levels of this protein class were found the most elevated in a Moroccan W8 accession with relatively high estimated total protein content (∼12% higher than average; Supplementary Table 2 and Supplementary Figure 3B). In some instances, the downregulation of the β-conglutin subfamilies were compensated for by an elevation in the other conglutin subfamilies. For example, in the low β-conglutin containing D3 cultivar, significantly higher levels of α 1-, α 2-, α 3-, β 1-, β 5-, β 7-, γ1-conglutin peptides were measured (Figures 3A,B,D), which contributed to the high total protein content measured for this cultivar (Supplementary Table 2).

#### Delta-Conglutin Protein Family

The four δ-conglutin subfamilies (δ1 to δ4) include 2S sulfur-rich albumin proteins, which belong to prolamin superfamily. This class of proteins in lupin are relatively small and are proposed to be mainly involved in storage function; however, they may also have a defense role in the seed due to the structural similarities with the plant cereal α-amylase/trypsin inhibitor family (40). The δ-conglutin fraction were also found to have low digestibility properties (41) and allergenicity potential (42, 43). Within this family the δ1 and δ3 sequences share a high sequence identity (over 98%), for which the discriminatory peptide (SSQESEESEELDQCCEQLNELNSQR, unique to δ1) and the shared peptides demonstrated similar trends and were employed for evaluation of these proteins over the study genotypes. Among the δ-conglutin subfamilies, substantial divergence was observed in the abundance of the δ2 and δ4 peptides where 83 and 116% overall variance were estimated for these proteins, respectively (Figure 3C). The δ2-conglutin peptides were found to be significantly increased in abundance in the wild accessions W26, W9, W26, and W10; on the contrary, these peptide markers were absent or present at low levels in 21 of the investigated genotypes (including 8 domesticated and 13 wild accessions). The δ4-conglutin is the most distinct protein within this family, the peptides monitored for which were comparably low in abundance and exhibited inconsistent trends. For ALQPVMEK and YCYSEAK peptides, this may be explained by genotype-specific amino acid substitution in these peptide regions. Overall, D5 and W18 are among the highest δ-conglutin containing accessions, whilst the lower levels were notable for D3 and W28 (Supplementary Figure 3C). The protein digestibility level study can determine if the genotypes with lower overall δ-conglutin levels have the potential to offer a higher nutritional quality for feed or food purposes.

#### Gamma-Conglutin Protein Family

This protein family has garnered special interest due to their key role in the lupin antihyperglycemic properties (44). Two γ-conglutin subfamilies are included in this protein class, which are basic 7S globulins and account for the least abundant fraction of conglutins in NLL seeds (37). Similar to other conglutin families, the γ-conglutins are also predominantly stored in the storage vacuoles; however, they are also found in the extracellular apoplectic regions and exhibit stability during germination, which suggests that these proteins may not fall in the classical category of the seed storage proteins (40). The aspartic-type endopeptidase activity GO term is assigned to γ-conglutins, which are also proposed to be involved in plant defense mechanisms (44). In terms of allergenicity, there is some level of discrepancy regarding the immunogenicity of these proteins ranging from weak to strong in different reports (38). Herein, the *in silico* epitope mapping evaluations revealed the presence of two overlapping epitopes from soybean 7S basic globulin 2 (UniProt ID: Q8RVH5) protein within the structure of the two γ-conglutins (Supplementary Figure 2C), which indicates the possible involvement of these proteins in the cross-allergic reactions with soybean.

The γ-conglutin sequences have relatively few tryptic cleavage sites and unlike the other conglutin families (α, β, and δ) were previously reported to be undetectable through the untargeted analysis of the Tris-HCl extracted lupin samples (45). Nevertheless, herein unique tryptic peptides corresponding to the two γ-conglutin subfamilies were determined and measured for the evaluation of these proteins. This indicates the resistance of γ-conglutins in native state to proteolytic degradation and highlights the ability of the denaturing extraction buffer used to assist in peptide liberation using trypsin digestion. Overall, a low variance was observed for γ-conglutins across the genotypes studied (14.2% for γ1 and 27.4% for γ2) (Figure 3D). Wherein, the relative abundances measured for the γ1-conglutin peptides were considerably higher compared to the γ2-conglutin peptide responses, a pattern which was also displayed in the RNA expression levels of the NLL γ-conglutins. The Portuguese wild accession (W14) with the highest protein estimate result was noted to have the highest overall γ-conglutin levels, whilst the Moroccan accession (W21) exhibited the lowest γ-conglutin content (Supplementary Figure 3D). The relative abundances of the γ1-conglutin peptides were significantly higher in D2-D5, and D7, whilst the γ2 specific peptides were found at significantly higher levels in the D4, D6, and D7 cultivars, this elevation may implicate the compensation for the suppressed β-conglutin levels across these domesticated lines, for ensuring the overall protein quantity within the seed, a phenomenon which has been previously reported for other seeds such as ultra-low-gluten barley cultivars (46) and transgenic soybean seeds (47). These NLL cultivars with lower allergenic β-conglutin content and higher bioactive γ-conglutin levels may offer enhanced health benefits; however, the lupin seed proteome also includes several thousand moderate or low abundant proteins (19). For deeper understanding of the proteome composition and rebalancing mechanisms a comprehensive investigation of the proteome-wide alterations is essential.

## Conclusion

Knowledge of the conglutin abundance diversity within NLL germplasm can be used to identify superior genotypes and facilitate the development of NLL varieties with optimal protein composition and traits. The targeted LC-MS/MS assay developed herein allowed the discrimination and quantitative evaluation of the conglutin proteins’ subclasses across a diverse range of domesticated and wild NLL accessions. Distinct differences were observed across the analyzed lines based on their conglutin profiles, wherein the major variability was associated with the β- and δ-conglutin protein contents. Importantly, the absence/lower abundance of the β2- to β6-conglutin subfamilies were noted across several Australian and Polish domesticated cultivars. This led to the suppression of the overall allergenic β-conglutin levels in these NLLs, for which some degree of compensatory elevation of α- and γ-conglutin families was noted. The identified potential hypoallergenic NLL varieties can be used for commercial cultivation or be exploited in breeding strategies to enhance the quality of lupin gain a food ingredient. Further studies focusing on the digestibility values and clinical immunogenicity of these lines can unravel the association of the conglutin profiles with nutritional quality of lupin varieties.

## Data Availability Statement

The datasets presented in this study have been uploaded to the CSIRO Data Access Portal. Data access link: https://doi.org/10.25919/pfkr-s130.

## Author Contributions

MC conceived the design of the study, provided guidance to AT through the sample preparation, data acquisition and processing steps. AT prepared the samples, analyzed, visualized the data, and drafted the manuscript. AJ provided AT guidance to build the lupin specific database, sequence analysis, epitope mapping, and data visualization. JB provided AT guidance to design the targeted MRM assay, develop scripts for data curation, statistical analysis, and visualization. MN-W provided assistance with the sample preparation and data acquisition. TL provided guidance to AT for sample preparation and targeted assay development. All authors reviewed and approved the final version of the manuscript.

## Conflict of Interest

The authors declare that the research was conducted in the absence of any commercial or financial relationships that could be construed as a potential conflict of interest.

## Publisher’s Note

All claims expressed in this article are solely those of the authors and do not necessarily represent those of their affiliated organizations, or those of the publisher, the editors and the reviewers. Any product that may be evaluated in this article, or claim that may be made by its manufacturer, is not guaranteed or endorsed by the publisher.

## Footnotes

1
www.lupinexpress.org

2
https://www.iedb.org/

3
https://software.broadinstitute.org/morpheus/

## Supplementary Material

Supplementary Data Sheet 1 Supporting figures and tables.

Supplementary Data Sheet 2 MRM assay transition list.

## Abbreviations

LC-MS: Liquid chromatography-mass spectrometry
LC-MRM-MS: Liquid chromatography-multiple reaction monitoring-mass spectrometry
NLL: Narrow-leafed lupin
DTT: dithiothreitol
PCA: Principal component analysis
HCA: Hierarchical cluster analysis
GO: Gene ontology
IDA: Information dependent acquisition.

## References

[1] McKinsey & Company. *Alternative Proteins: The Race for Market Share is On.* (2019). Available online at: https://www.mckinsey.com/industries/agriculture/our-insights/alternative-proteins-the-race-for-market-share-is-on (accessed December 22, 2021)

[2] Henchion M Hayes M Mullen AM Fenelon M Tiwari B. Future protein supply and demand: strategies and factors influencing a sustainable equilibrium. *Foods.* (2017) 6:53. 10.3390/foods6070053 28726744 PMC5532560

[3] Marinangeli CPF Curran J Barr SI Slavin J Puri S Swaminathan S Enhancing nutrition with pulses: defining a recommended serving size for adults. *Nutr Rev.* (2017) 75:990–1006. 10.1093/nutrit/nux058 29202192 PMC5914352

[4] Arnoldi A Boschin G Zanoni C Lammi C. The health benefits of sweet lupin seed flours and isolated proteins. *J Funct Foods.* (2015) 18:550–63. 10.1016/j.jff.2015.08.012

[5] Berger JD Buirchell BJ Luckett DJ Nelson MN. Domestication bottlenecks limit genetic diversity and constrain adaptation in narrow-leafed lupin (*Lupinus angustifolius L.*). *Theor Appl Genet.* (2012) 124:637–52. 10.1007/s00122-011-1736-z 22069118

[6] Kamphuis LG Garg G Foley R Singh KB. Genomic resources for lupins are coming of age. *Legum Sci.* (2021) 3:e77. 10.1002/leg3.77

[7] Foley RC Jimenez-Lopez JC Kamphuis LG Hane JK Melser S Singh KB. Analysis of conglutin seed storage proteins across lupin species using transcriptomic, protein and comparative genomic approaches. *BMC Plant Biol.* (2015) 15:106. 10.1186/s12870-015-0485-6 25902794 PMC4407355

[8] Tapadia M Johnson S Utikar R Newsholme P Carlessi R. Antidiabetic effects and mechanisms of action of γ-conglutin from lupin seeds. *J Funct Foods.* (2021) 87:104786. 10.1016/j.jff.2021.104786

[9] Jimenez-Lopez JC. Narrow-leafed lupin (*Lupinus angustifolius L.*) β-conglutin: a multifunctional family of proteins with roles in plant defence, human health benefits, and potential uses as functional food. *Legum Sci.* (2020) 2:e33. 10.1002/leg3.33

[10] Mazumder K Biswas B Kerr PG Blanchard C Nabila A Golder M Comparative assessment of nutritional, thermal, rheological and functional properties of nine Australian lupin cultivars. *Sci Rep.* (2021) 11:21515. 10.1038/s41598-021-00838-x 34728683 PMC8564527

[11] Bettzieche A Brandsch C Schmidt M Weisse K Eder K Stangl GI. Differing effect of protein isolates from different cultivars of blue lupin on plasma lipoproteins of hypercholesterolemic rats. *Biosci Biotechnol Biochem.* (2008) 72:3114–21. 10.1271/bbb.80221 19060384

[12] Jimenez-Lopez JC Foley RC Brear E Clarke VC Lima-Cabello E Florido JF Characterization of narrow-leaf lupin (*Lupinus angustifolius L.*) recombinant major allergen IgE-binding proteins and the natural beta-conglutin counterparts in sweet lupin seed species. *Food Chem.* (2018) 244:60–70. 10.1016/j.foodchem.2017.10.015 29120805

[13] Tomczak A Zielinska-Dawidziak M Piasecka-Kwiatkowska D Springer E Lampart-Szczapa E. Cross-reactions between proteins isolated from new narrow-leafed lupine breeding lines and antibodies present in the sera of patients sensitized to soybeans and peanuts. *Eur Food Res Technol.* (2019) 245:433–41. 10.1007/s00217-018-3175-4

[14] Tai HH Bush RS. Analysis of lupin seed protein digestibility using gel electrophoresis and immunoblots. *J Anim Sci.* (1997) 75:1934–40. 10.2527/1997.7571934x 9222852

[15] Islam S Ma W Ma J Buirchell BJ Appels R Yan G. Diversity of seed protein among the Australian narrow-leafed lupin (*Lupinus angustifolius L.*) cultivars. *Crop Pasture Sci.* (2011) 62:765–75. 10.1071/CP11046

[16] Islam S Yan G Appels R Ma W. Comparative proteome analysis of seed storage and allergenic proteins among four narrow-leafed lupin cultivars. *Food Chem.* (2012) 135:1230–8. 10.1016/j.foodchem.2012.05.081 22953848

[17] Taylor CM Kamphuis LG Zhang W Garg G Berger JD Mousavi-Derazmahalleh M INDEL variation in the regulatory region of the major flowering time gene LanFTc1 is associated with vernalization response and flowering time in narrow-leafed lupin (Lupinus angustifolius L.). *Plant Cell Environ.* (2019) 42:174–87. 10.1111/pce.13320 29677403 PMC7379684

[18] Mousavi-Derazmahalleh M Bayer PE Nevado B Hurgobin B Filatov D Kilian A Exploring the genetic and adaptive diversity of a pan-Mediterranean crop wild relative: narrow-leafed lupin. *Theor Appl Genet.* (2018) 131:887–901. 10.1007/s00122-017-3045-7 29353413 PMC5852200

[19] Tahmasian A Broadbent JA Juhász A Nye-Wood M Le TT Bose U Evaluation of protein extraction methods for in-depth proteome analysis of narrow-leafed lupin (*Lupinus angustifolius*) seeds. *Food Chem.* (2022) 367:130722. 10.1016/j.foodchem.2021.130722 34375893

[20] Bose U Broadbent JA Juhász A Karnaneedi S Johnston EB Stockwell S Protein extraction protocols for optimal proteome measurement and arginine kinase quantitation from cricket *Acheta domesticus* for food safety assessment. *Food Chem.* (2021) 348:129110. 10.1016/j.foodchem.2021.129110 33508605

[21] Colgrave ML Byrne K Howitt CA. Liquid chromatography-mass spectrometry analysis reveals hydrolyzed gluten in beers crafted To remove gluten. *J Agric Food Chem.* (2017) 65:9715–25. 10.1021/acs.jafc.7b03742 29047268

[22] Kamphuis LG Hane JK Nelson MN Gao L Atkins CA Singh KB. Transcriptome sequencing of different narrow-leafed lupin tissue types provides a comprehensive uni-gene assembly and extensive gene-based molecular markers. *Plant Biotech J.* (2015) 13:14–25. 10.1111/pbi.12229 25060816 PMC4309465

[23] Bateman A Coin L Durbin R Finn RD Hollich V Griffiths-Jones S The Pfam protein families database. *Nucleic Acids Res.* (2004) 32:D138–41. 10.1093/nar/gkh121 14681378 PMC308855

[24] Larkin MA Blackshields G Brown NP Chenna R McGettigan PA McWilliam H Clustal W and Clustal X version 2.0. *Bioinformatics.* (2007) 23:2947–8. 10.1093/bioinformatics/btm404 17846036

[25] Shilov IV Seymour SL Patel AA Loboda A Tang WH Keating SP The Paragon Algorithm, a next generation search engine that uses sequence temperature values and feature probabilities to identify peptides from tandem mass spectra. *Mol Cell Proteomics.* (2007) 6:1638–55. 10.1074/mcp.T600050-MCP200 17533153

[26] Tang WH Shilov IV Seymour SL. Nonlinear fitting method for determining local false discovery rates from decoy database searches. *J Proteome Res.* (2008) 7:3661–7. 10.1021/pr070492f 18700793

[27] Chen C Li Z Huang H Suzek BE Wu CH UniProt C. A fast peptide match service for UniProt knowledgebase. *Bioinformatics.* (2013) 29:2808–9. 10.1093/bioinformatics/btt484 23958731 PMC3799477

[28] MacLean B Tomazela DM Shulman N Chambers M Finney GL Frewen B Skyline: an open source document editor for creating and analyzing targeted proteomics experiments. *Bioinformatics.* (2010) 26:966–8. 10.1093/bioinformatics/btq054 20147306 PMC2844992

[29] Ritchie ME Phipson B Wu D Hu Y Law CW Shi W Limma powers differential expression analyses for RNA-sequencing and microarray studies. *Nucleic Acids Res.* (2015) 43:e47. 10.1093/nar/gkv007 25605792 PMC4402510

[30] Lee YP Mori TA Sipsas S Barden A Puddey IB Burke V Lupin-enriched bread increases satiety and reduces energy intake acutely. *Am J Clin Nutr.* (2006) 84:975–80. 10.1093/ajcn/84.5.975 17093146

[31] Monteiro S Freitas R Rajasekhar BT Teixeira AR Ferreira RB. The unique biosynthetic route from lupinus β-conglutin gene to blad. *PLoS One.* (2010) 5:e8542. 10.1371/journal.pone.0008542 20066045 PMC2798717

[32] Monaci L Pilolli R De Angelis E Crespo JF Novak N Cabanillas B. Food allergens: classification, molecular properties, characterization, and detection in food sources. In: Toldrá F editor. *Advances in Food and Nutrition Research.* Cambridge, MA: Academic Press (2020). p. 113–46. 10.1016/bs.afnr.2020.03.001 32711861

[33] Lima-Cabello E Robles-Bolivar P Alché JD Jimenez-Lopez JC. Narrow leafed lupin beta-conglutin proteins epitopes identification and molecular features analysis involved in cross-allergenicity to peanut and other legumes. *Genomics Comput Biol.* (2016) 2:e29. 10.18547/gcb.2016.vol2.iss1.e29

[34] Mousavi-Derazmahalleh M Nevado B Bayer PE Filatov DA Hane JK Edwards D The western Mediterranean region provided the founder population of domesticated narrow-leafed lupin. *Theor Appl Genet.* (2018) 131:2543–54. 10.1007/s00122-018-3171-x 30225643 PMC6244526

[35] Cowling WA. *Pedigrees and Characteristics of Narrow-Leafed Lupin Cultivators Released in Australia from 1967 to 1998 by Wallace .A. Cowling.* Western Australia: Agriculture Western Australia (1999). 11 p.

[36] Cowling WA. Genetic diversity in narrow-leafed lupin breeding after the domestication bottleneck. In: Singh KB Kamphuis LG Nelson MN editors. *The Lupin Genome. Compendium Plant Genomes.* Cham: Springer (2020). p. 1–17. 10.1007/978-3-030-21270-4

[37] Foley RC Gao LL Spriggs A Soo LYC Goggin DE Smith PMC Identification and characterisation of seed storage protein transcripts from *Lupinus angustifolius*. *BMC Plant Biol.* (2011) 11:59. 10.1186/1471-2229-11-59 21457583 PMC3078879

[38] Villa C Costa J Mafra I. Lupine allergens: clinical relevance, molecular characterization, cross−reactivity, and detection strategies. *Compr Rev Food Sci Food Saf.* (2020) 19:3886–915. 10.1111/1541-4337.12646 33337069

[39] Hufnagel B Marques A Soriano A Marquès L Divol F Doumas P High-quality genome sequence of white lupin provides insight into soil exploration and seed quality. *Nat Commun.* (2020) 11:492. 10.1038/s41467-019-14197-9 31980615 PMC6981116

[40] Duranti M Consonni A Magni C Sessa F Scarafoni A. The major proteins of lupin seed: characterisation and molecular properties for use as functional and nutraceutical ingredients. *Trends Food Sci Technol.* (2008) 19:624–33. 10.1016/j.tifs.2008.07.002

[41] Ogura T Hernández A Aizawa T Ogihara J Sunairi M Alcaino J Identification of a low digestibility δ-Conglutin in yellow lupin (*Lupinus luteus L.*) seed meal for atlantic salmon (*Salmo salar L.*) by coupling 2D-PAGE and mass spectrometry. *PLoS One.* (2013) 8:e80369. 10.1371/journal.pone.0080369 24278278 PMC3838402

[42] Dooper MM Plassen C Holden L Lindvik H Faeste CK. Immunoglobulin E cross-reactivity between lupine conglutins and peanut allergens in serum of lupine-allergic individuals. *J Investig Allergol Clin Immunol.* (2009) 19:283–91. 19639724

[43] Holden L Sletten GB Lindvik H Faeste CK Dooper MM. Characterization of IgE binding to lupin, peanut and almond with sera from lupin-allergic patients. *Int Arch Allergy Immunol.* (2008) 146:267–76. 10.1159/000121461 18362472

[44] Mane SP Johnson SK Duranti M Pareek VK Utikar RP. Lupin seed γ-conglutin: extraction and purification methods - A review. *Trends Food Sci Technol.* (2018) 73:1–11. 10.1016/j.tifs.2017.12.008

[45] Aiello G Li Y Boschin G Stanziale M Lammi C Arnoldi A. Analysis of narrow-leaf lupin proteins in lupin-enriched pasta by untargeted and targeted mass spectrometry. *Foods.* (2020) 9:1083. 10.3390/foods908108 PMC7465979 32784441

[46] Bose U Broadbent JA Byrne K Blundell MJ Howitt CA Colgrave ML. Proteome analysis of hordein-null barley lines reveals storage protein synthesis and compensation mechanisms. *J Agric Food Chem.* (2020) 68:5763–75. 10.1021/acs.jafc.0c01410 32374605

[47] Kinney AJ Jung R Herman EM. Cosuppression of the alpha subunits of beta-conglycinin in transgenic soybean seeds induces the formation of endoplasmic reticulum-derived protein bodies. *Plant Cell.* (2001) 13:1165–78. 10.1105/tpc.13.5.1165 11340189 PMC135556

