# Supplementary material for: Evaluation of the Major Seed Storage Proteins, the Conglutins, Across Genetically Diverse Narrow-Leafed Lupin Varieties
Source: Front Nutr. 2022 May 13;9:842168. doi: 10.3389/fnut.2022.842168 (PMC9136412; doi:10.3389/fnut.2022.842168)
Supplement: Supplementary Data Sheet 1 — Supporting figures and tables. [file Data_Sheet_1.docx]

**Table S1.** Country of origin information, domestic/wild status, and the identifiers given to the 46 narrow-leafed lupin accessions used for this study.

| **Accession number** | **Accession name** | **Identifier** | **Origin** | **Domestic/wild^a^** | **Seed supplier^b^** |
| --- | --- | --- | --- | --- | --- |
| P29086 | Mandelup | D1 | Australia | d | AGT |
| P28806 | Bolero | D2 | Poland | d | CSIRO |
| P25795 | Emir | D3 | Poland | d | AGG |
| P26975 | Yorrel | D4 | Australia | d | AGG |
| P28324 | Kalya | D5 | Australia | d | AGT |
| P24138 | Marri | D6 | Australia | d | AGG |
| P28578 | Tanjil | D7 | Australia | d | CSIRO |
| P30000 | 83A:476 | D8 | Australia | d | CSIRO |
|  | Moonah | D9 | Australia | d | AGG |
| P20672 | Uniwhite | D10 | Australia | d | AGG |
| P28364 | Quilinock | D11 | Australia | d | AGG |
| P20681 | Unicrop | D12 | Australia | d | AGG |
| P28137 | Myallie | D13 | Australia | d | AGG |
| P28317 | Sonet | D14 | Poland | d | CSIRO |
|  | PBA Jurien | D15 | Australia | d | AGT |
| P29039 | BSKHA-640 | D16 | Belarus | d | CSIRO |
| P20720 | G111 | W1 | Italy | w | AGG |
| P22839 | GP051 | W2 | Portugal | w | AGG |
| P22831 | GP021 | W3 | Portugal | w | AGG |
| P22687 | GS037 | W4 | Spain | w | AGG |
| P22744 | GS123 | W5 | Spain | w | AGG |
| P22810 | NS009 | W6 | Spain | w | AGG |
| P25016 | QS212 | W7 | Spain | w | AGG |
| P27895 | MAR6783A | W8 | Morocco | w | AGG |
| P28195 | ANO3 | W9 | Algeria | w | AGG |
| P27436 | SYR6259A | W10 | Syria | w | CSIRO |
| P26603 | GRC5054A | W11 | Greece | w | AGG |
| P21624 | 1111.3 | W12 | Turkey | w | AGG |
| P22872 | GM120 | W13 | Morocco | w | AGG |
| P26297 | G84-159 | W14 | Portugal | w | CSIRO |
| P26239 | G84-091 | W15 | Spain | w | AGG |
| P25136 | NS030 | W16 | Spain | w | AGG |
| P26464 | GRC5011A | W17 | Greece | w | AGG |
| P28038 | MJS373 | W18 | Greece | w | CSIRO |
| P26423 | LO-1756 | W19 | Spain | w | CSIRO |
| P26167 | G84038 | W20 | Spain | w | AGG |
| P27893 | MAR6781A | W21 | Morocco | w | AGG |
| P26676 | CY06 | W22 | Cyprus | w | AGG |
| P26559 | GRC5038A | W23 | Greece | w | AGG |
| P27221 | MAR6009A | W24 | Morocco | w | AGG |
| P26668 | ITA5269A | W25 | Italy | w | AGG |
| P26625 | GRC5066A | W26 | Greece | w | AGG |
| P26170 | G84040 | W27 | Spain | w | CSIRO |
| P27255 | MAR6045A | W28 | Morocco | w | AGG |
| P25055 | GF001 | W29 | France | w | AGG |
| P22660 | Q031 | W30 | Israel | w | AGG |

^a^ w = wild and d = domesticated accessions.

^b^ AGG: Australian Grains Genebank, AGT: Australian Grain Technologies, CSIRO: CSIRO Agriculture and Food.

**Table S2.** The summary of the protein and peptide identifications (at 1% FDR) and the BCA protein estimation for each sample. The protein estimation data is shown as mean ± S.D. (CV%) (n=3 replicates).

| Identifier | Protein | Peptide | Protein estimation (mg/ml) |
| --- | --- | --- | --- |
| D1 | 1,994 | 8,812 | 6.28 ± 0.54 (8.64%) |
| D2 | 1,815 | 7,881 | 6.16 ± 0.34 (5.53%) |
| D3 | 1,694 | 7,991 | 8.82 ± 0.42 (4.79%) |
| D4 | 1,824 | 7,918 | 6.01 ± 0.68 (11.37%) |
| D5 | 1,831 | 8,125 | 7.1 ± 0.36 (5.09%) |
| D6 | 1,841 | 8,057 | 6.09 ± 0.55 (8.99%) |
| D7 | 1,589 | 7,098 | 7.38 ± 0.1 (1.29%) |
| D8 | 1,610 | 7,203 | 6.99 ± 0.9 (12.82%) |
| D9 | 1,879 | 8,012 | 7.04 ± 0.37 (5.2%) |
| D10 | 1,821 | 7,996 | 6.83 ± 0.55 (8.04%) |
| D11 | 1,773 | 8,321 | 7.25 ± 0.21 (2.84%) |
| D12 | 1,488 | 6,852 | 7.48 ± 0.46 (6.18%) |
| D13 | 1,929 | 8,813 | 8.6 ± 0.2 (2.29%) |
| D14 | 1,528 | 6,943 | 4.36 ± 0.5 (11.57%) |
| D15 | 1,461 | 10,113 | 8.96 ± 0.39 (4.3%) |
| D16 | 1,818 | 7,910 | 6.43 ± 0.48 (7.39%) |
| W1 | 1,824 | 8,274 | 6.35 ± 0.65 (10.28%) |
| W2 | 1,826 | 7,963 | 7.19 ± 0.11 (1.48%) |
| W3 | 1,811 | 8,071 | 8.24 ± 0.25 (3.08%) |
| W4 | 1,809 | 7,982 | 7.75 ± 0.11 (1.4%) |
| W5 | 1,678 | 7,530 | 7.1 ± 0.11 (1.56%) |
| W6 | 1,897 | 8,275 | 6.7 ± 0.59 (8.75%) |
| W7 | 1,692 | 7,513 | 6.74 ± 0.21 (3.17%) |
| W8 | 1,600 | 7,199 | 8.05 ± 0.21 (2.64%) |
| W9 | 1,687 | 7,880 | 7.15 ± 0.45 (6.24%) |
| W10 | 1,618 | 7,201 | 6.3 ± 0.9 (14.35%) |
| W11 | 1,820 | 8,300 | 7.63 ± 0.45 (5.9%) |
| W12 | 1,749 | 7,837 | 7.66 ± 0.73 (9.57%) |
| W13 | 1,621 | 7,573 | 7.8 ± 0.61 (7.77%) |
| W14 | 1,641 | 7,336 | 9.49 ± 1.57 (16.59%) |
| W15 | 1,549 | 7,251 | 7 ± 0.38 (5.42%) |
| W16 | 1,747 | 7,752 | 7.27 ± 0.18 (2.52%) |
| W17 | 1,735 | 7,640 | 7.99 ± 0.71 (8.94%) |
| W18 | 1,602 | 7,098 | 8.61 ± 0.34 (3.91%) |
| W19 | 1,520 | 6,645 | 7.25 ± 0.48 (6.65%) |
| W20 | 1,773 | 7,857 | 7.39 ± 0.89 (12%) |
| W21 | 1,734 | 7,705 | 7.05 ± 0.9 (12.72%) |
| W22 | 1,668 | 7,256 | 7.63 ± 0.34 (4.41%) |
| W23 | 1,747 | 7,905 | 7.72 ± 1.15 (14.87%) |
| W24 | 1,811 | 7,784 | 6.33 ± 0.62 (9.81%) |
| W25 | 1,987 | 8,417 | 6.55 ± 0.96 (14.65%) |
| W26 | 1,818 | 8,003 | 7.65 ± 0.96 (12.59%) |
| W27 | 1,598 | 7,296 | 5.57 ± 0.12 (2.25%) |
| W28 | 1,418 | 6,508 | 7.58 ± 0.45 (5.96%) |
| W29 | 1,784 | 7,919 | 6.25 ± 0.11 (1.76%) |
| W30 | 1,975 | 8,472 | 6.47 ± 0.27 (4.23%) |
| combined all | 3,534 | 20,371 |  |

**
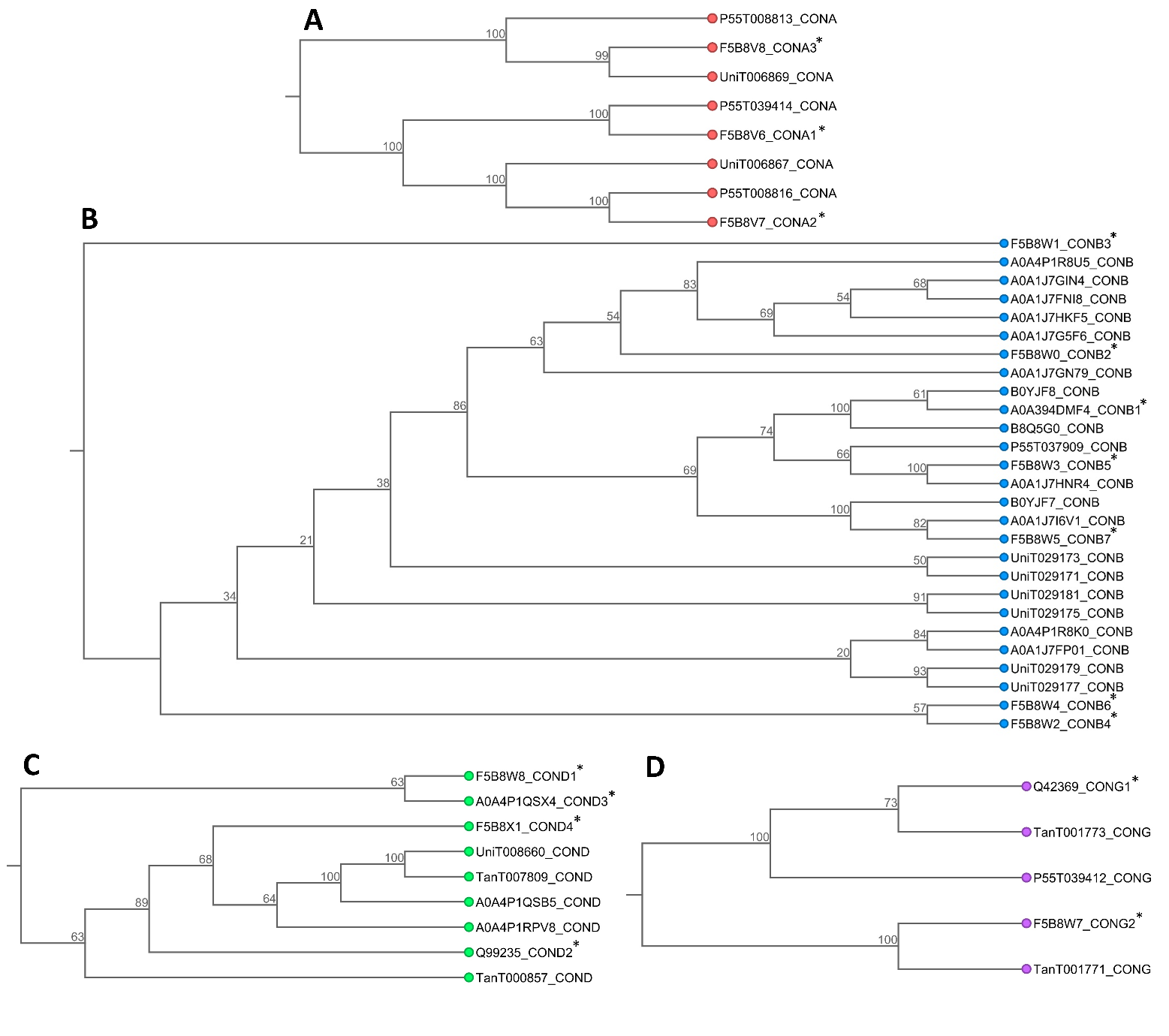
**

**Figure S1.** The phylogenetic relationships of the α (A), β (B), δ (C), and γ (D) conglutin protein sequences identified from NLL database used for the study (the 16 known conglutins protein accessions are indicated with an asterisk).

**Table S3.** Summary of the quantitative peptides measured by LC-MRM-MS, for monitoring conglutin proteins across 46 NLL genotypes. The intensity of the peptides is represented as the average peak area across the analysed lines and the corresponding technical variation (related to sample preparation and analysis) is depicted as the average of the CV% values calculated for the replicates of each genotype.

| **Conglutin**  **family** | **Conglutin sub-family** | **Peptide search result** | **Peptide** | **Average peak area** | **Average CV%** |
| --- | --- | --- | --- | --- | --- |
| **α** | α1 | F5B8V6^*^  P55T039414 | ALPLDVVAHAFNLDR | 50,748,283.3 | 7.5 |
|  |  |  | EGSIVEVK | 160,935,297.8 | 5.4 |
|  |  |  | ETYEEPQEQEQGQGPRPQDR | 132,914,731.4 | 5.3 |
|  |  |  | FYLSGNQEQEFLQYQQK | 11,566,171.8 | 5.2 |
|  |  |  | GIFGLIFPGCR | 451,257,103.2 | 3 |
|  |  |  | HNIGQSTSPDAYNPQAGR | 94,400,761.5 | 2.9 |
|  |  |  | QQPQQNECQFQR | 118,987,705.6 | 5.1 |
|  |  |  | SEAGTIETWNPNNDQLR | 72,081,723.5 | 4.8 |
|  |  |  | SLDDNFSYVAFK | 160,413,929.9 | 2.7 |
|  |  |  | TLTSLDFPILR | 458,104,241.9 | 2.6 |
|  |  |  | WLGLAAEHGSIYK | 62,179,567.2 | 4.4 |
|  | α2 | F5B8V7^*^  P55T008816  UniT006867 | ADLYNPNAGR | 343,982,110.8 | 3.8 |
|  |  |  | GLSIISPK | 560,441,414.8 | 3.2 |
|  |  |  | ISSVNSLTLPILR | 489,296,642.2 | 2.6 |
|  |  |  | TNDLAATSPVK | 346,707,284.5 | 3.1 |
|  | α3 | F5B8V8^*^ | ADLYNPTAGR | 230,017,444.4 | 3.8 |
|  |  |  | FNECQLDR | 90,692,752.4 | 3.9 |
|  |  |  | FYLAGNPEEEYPETQQQR | 45,209,482.7 | 5 |
|  |  |  | GIPAEVLANAFR | 457,756,545.3 | 2.5 |
|  |  |  | IESEGGVTETWNSNKPELR | 183,599,729.1 | 4.3 |
|  |  |  | LSLNQVSELK | 155,260,066.3 | 4.7 |
|  |  |  | TNDQATTSPLK | 143,550,060.7 | 3.3 |
| **β** | β1 | F5B8V9^*^/A0A394DMF4^*^  B8Q5G0  B0YJF8 | FQTYYR | 154,355,999.7 | 5.7 |
|  |  |  | HAQSSSGEGKPSESGPFNLR | 16,483,596.7 | 4 |
|  |  |  | IIEFQSKPNTLILPK | 207,246,557.4 | 4.5 |
|  |  |  | LYDFYPSTTK | 149,334,575.2 | 3.5 |
|  |  |  | NPYHFSSNR | 26,770,690.3 | 3.7 |
|  |  |  | YEEIER | 283,176,856.1 | 6.8 |
|  |  |  | HDGEPSR | 3,854,723.1 | 7.5 |
|  | β2 | F5B8W0^*^ | EQEQGSSSSSGR | 1,389,567.4 | 10.8 |
|  |  |  | EQQQEQDSR | 1,519,346.6 | 12.1 |
|  |  |  | FQTLYK | 188,805,886.2 | 4.2 |
|  |  |  | NPYYFSYER | 157,911,007.8 | 3.2 |
|  |  | F5B8W0^*^  A0A1J7FNI8  A0A1J7G5F6  A0A1J7GN79 | QLTFPGSVEDVER | 245,512,866.2 | 4 |
|  |  |  | QAYNLEHGDALR | 103,965,296.3 | 5.9 |
|  |  |  | IILGNEDGQEDEEQSR | 28,421,387.0 | 8 |
|  |  |  | SDEPIYSNK | 78,021,941.2 | 7 |
|  | β3 | F5B8W1^*^ | ESEESQEEER | 3,041,133.7 | 12.4 |
|  |  |  | GKPYESGPFNLR | 61,996,952.9 | 8 |
|  |  |  | GLIFPGSAEDVER | 574,637,235 | 26.1 |
|  | β4,6 | F5B8W2^*^ | GLTFPGSTEDVER | 367,213,616.1 | 8.1 |
|  |  |  | QLDTEVK | 159,728,858.1 | 11.7 |
|  |  | F5B8W4^*^ | QSAYER | 12,772,482.2 | 20.5 |
|  |  | F5B8W2^*^  F5B8W4^*^ | FGNFYEITPNR | 230,615,114.5 | 10.9 |
|  |  |  | ILLGNEDEQEDDEQR | 22,094,735.7 | 12 |
|  | β5 | F5B8W3^*^ | NTLEATFNTHYEEIQR | 79,973,783 | 9.8 |
|  |  | F5B8W3^*^  A0A1J7HNR4 | ELIFPGSAEDVER | 139,537,926.2 | 3.8 |
|  |  |  | EQEQSHQDEGVIVR | 3,583,685.7 | 7.8 |
|  |  |  | LSEGDILVIPAGHPLSINASSNLR | 14,925,656.1 | 9.4 |
|  |  |  | QQDEQEVEEVR | 44,616,867.7 | 5 |
|  | β7 | F5B8W5^*^  B0YJF7 | LAIPINNPSNFYDFYPSSTK | 8,359,639.7 | 6.2 |
|  |  | F5B8W5^*^  A0A1J7I6V1  B0YJF7 | ELTFPGSAQDVER | 75,195,601.5 | 5.6 |
|  |  |  | GQEQSYQDEGVIVR | 9,904,282.5 | 6.9 |
|  |  |  | NPYHFSSER | 6,838,836.7 | 6 |
|  |  |  | \| NQQQSYFANAQPQQK \| \| --- \| | 3,714,421.5 | 7.3 |
|  | β | A0A1J7HKF5 | AIFVVVVDK | 8,332,629.1 | 33.9 |
|  |  |  | ELTFPGSVEDVER | 100,807,513.3 | 5.6 |
|  |  | A0A1J7FP01 | QQPRPHLQEEQER | 1,121,134.2 | 16 |
|  |  | A0A4P1R8U5 A0A1J7GIN4 | SHQEEGVIVR | 58,171.3 | 18.2 |
| **δ** | δ1,3 | F5B8W8^*^ | SSQESEESEELDQCCEQLNELNSQR | 24,604,251.8 | 6.5 |
|  |  | F5B8X0^*^  A0A4P1QSX4  F5B8W8^*^ | ALQQIYESQSEQCEGR | 223,722,880.5 | 3.5 |
|  |  |  | CNINPDEE | 46,325,758.8 | 5 |
|  |  |  | HCENHIDQR | 21,285,867.8 | 5.2 |
|  |  |  | QQEQQLEGELEK | 149,913,032.8 | 3.4 |
|  | δ2 | Q99235^*^ | ALQQIYESQSEQCEGSQQEQQLEQELEK | 3,119,081.2 | 7.4 |
|  |  |  | CDVNPDEE | 3,627,717.3 | 10.1 |
|  |  |  | HCENHIAQR | 18,560,740.4 | 7.3 |
|  |  |  | SSQEYSEESEELDQCCEQLNELNSQR | 3,033,531.7 | 8 |
|  | δ4 | F5B8X1^*^ | ALQPVMEK | 30,072,347.1 | 6.8 |
|  |  |  | HQEDCFPR | 130,136.6 | 10.8 |
|  |  |  | LGQCCEILSDLSEGCQCR | 5,417,757.1 | 5.7 |
|  |  |  | YCYSEAK | 142,671.3 | 9.8 |
| **γ** | γ2 | F5B8W7^*^ | TPLMQVPVLLDLNGK | 3,683,829.9 | 9.5 |
|  |  | A0A4P1RSN4  F5B8W7^*^ | QFTMCLSR | 3,488,022.8 | 7.3 |
|  |  |  | TCANLFDLNNA | 3,232,892.6 | 3.4 |
|  |  |  | VEFNSNPLK | 5,695,686.8 | 7.4 |
|  | γ1 | Q42369^*^ | HLWVTCSQHYSSSTYQAPFCHSTQCSR | 1,245,961.9 | 8.1 |
|  |  |  | ISGGAPSVDLILDK | 133,388,344.4 | 4.9 |
|  |  |  | NDAVWR | 92,255,233 | 5.4 |
|  |  |  | QGEYFIQVNAIR | 123,292,446.6 | 4.9 |
|  |  |  | VGFNSNSLK | 105,195,431.6 | 6.6 |
|  |  |  | VPQFLFSCAPSFLAQK | 45,970,057.8 | 8.2 |

^*^Indicates the known conglutin protein accessions.

**
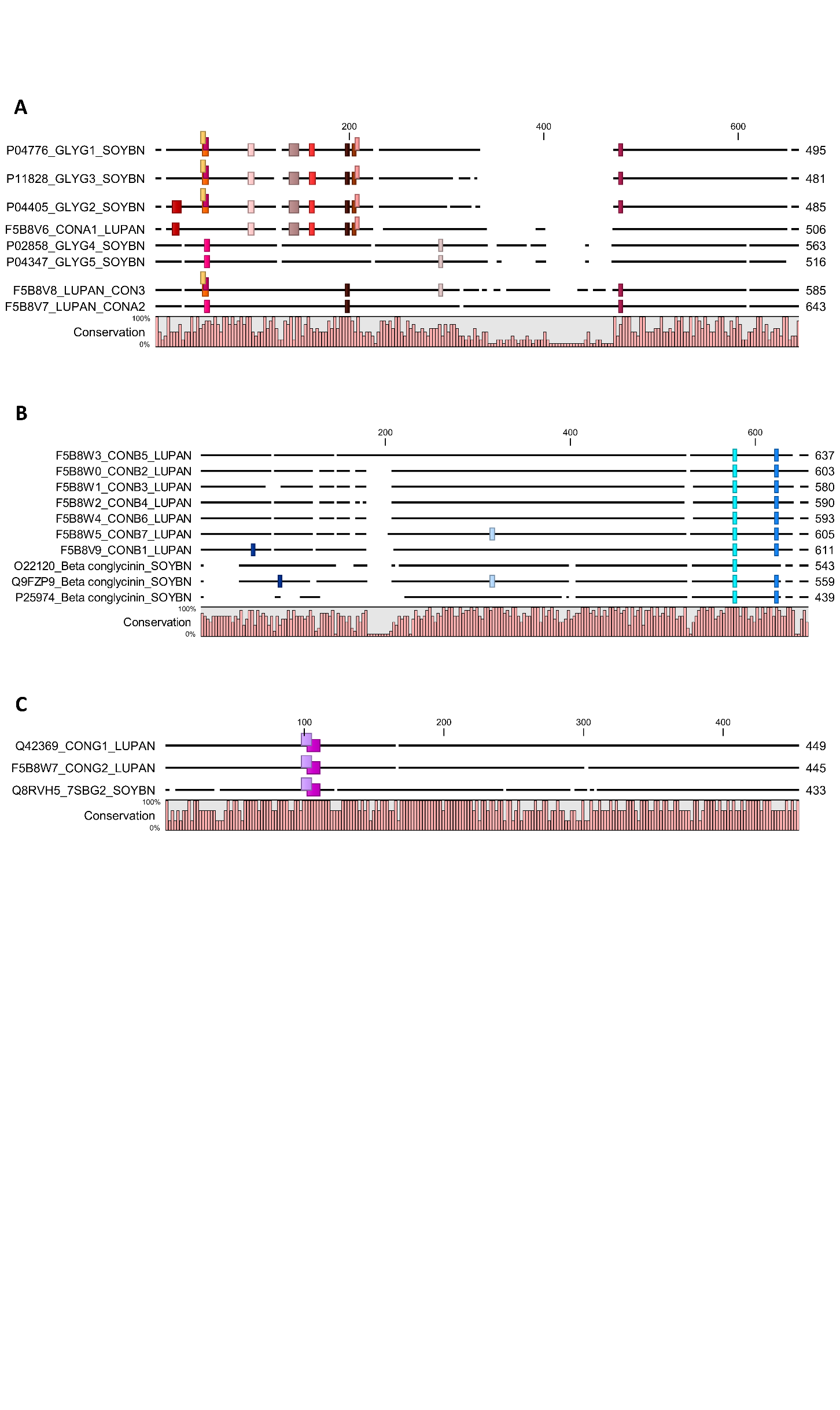
**

**Figure S2.** The sequence alignment of the lupin α (A), β (B), and γ (C) conglutin sequences with the homologous allergenic proteins from soybean. The coloured blocks represent the known linear epitopes (retrieved from IEDB database) of the soybean Gly m 6, Gly m 5 allergens and 7S basic globulin 2 protein found with 100% sequence identity in the α (A), β (B), and γ (C) conglutin sequences, respectively.

**
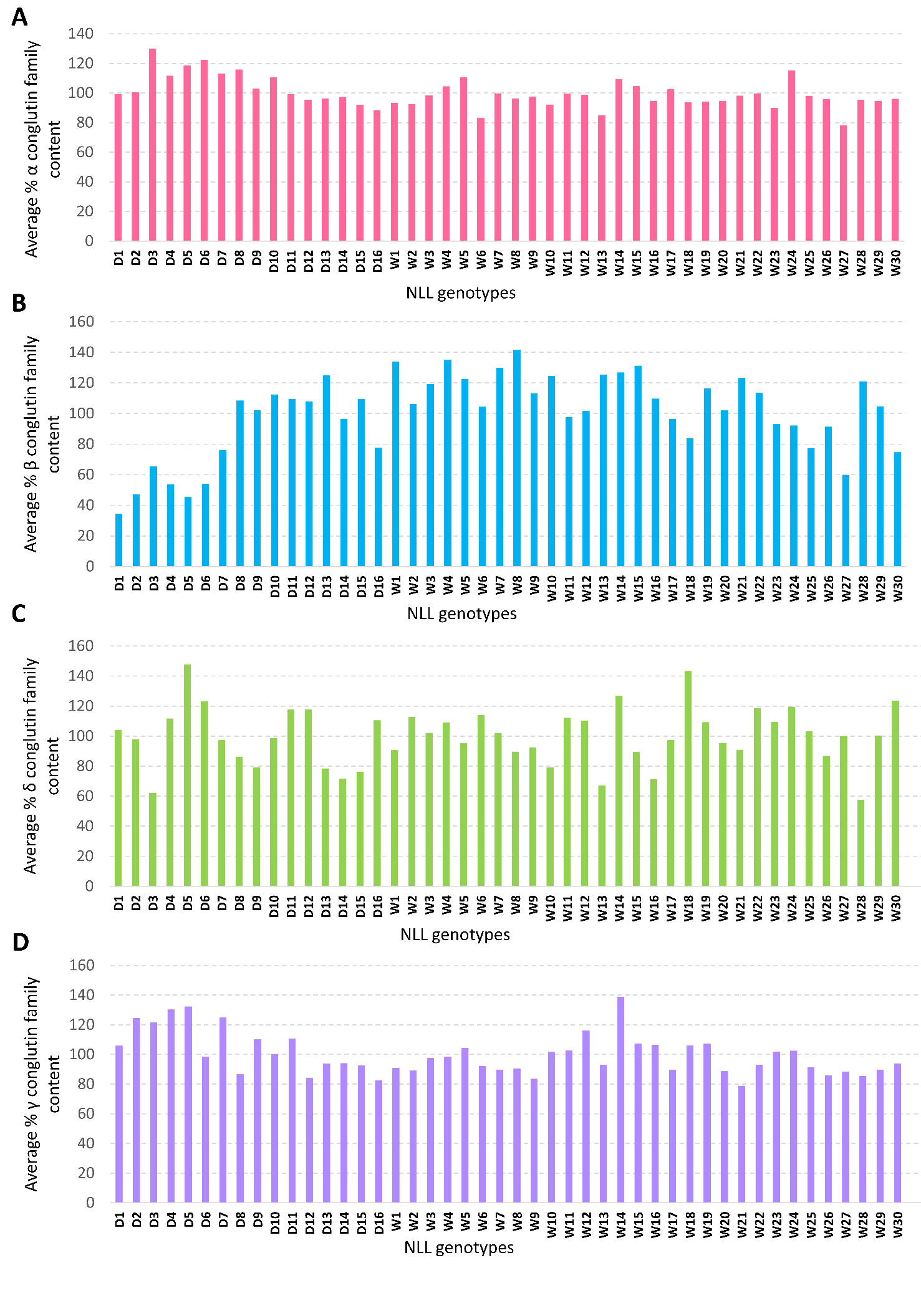
**

**Figure S3.** Average percentage of the total α- (A), β- (B), δ- (C) and γ- (D) conglutin contents across the 46 NLL genotypes.
